# Thiopurine Methyltransferase Predicts the Extent of Cytotoxicty and DNA Damage in Astroglial Cells after Thioguanine Exposure

**DOI:** 10.1371/journal.pone.0029163

**Published:** 2011-12-21

**Authors:** Amira Hosni-Ahmed, Joseph D. Barnes, Jim Wan, Terreia S. Jones

**Affiliations:** 1 Department of Clinical Pharmacy, University of Tennessee Health Science Center, Memphis, Tennessee, United States of America; 2 Department of Pharmaceutical Sciences, University of Tennessee Health Science Center, Memphis, Tennessee, United States of America; 3 Department of Neurosurgery, University of Tennessee Health Science Center, Memphis, Tennessee, United States of America; 4 Division of Biostatistics and Epidemiology, University of Tennessee Health Science Center, Memphis, Tennessee, United States of America; 5 Department of Chemistry, College of Science, Fayoum University, Fayoum, Egypt; Dr. Margarete Fischer-Bosch Institute of Clinical Pharmacology, Germany

## Abstract

Thiopurine methyltransferase (Tpmt) is the primary enzyme responsible for deactivating thiopurine drugs. Thiopurine drugs (i.e., thioguanine [TG], mercaptopurine, azathioprine) are commonly used for the treatment of cancer, organ transplant, and autoimmune disorders. Chronic thiopurine therapy has been linked to the development of brain cancer (most commonly astrocytomas), and Tpmt status has been associated with this risk. Therefore, we investigated whether the level of Tpmt protein activity could predict TG-associated cytotoxicity and DNA damage in astrocytic cells. We found that TG induced cytotoxicity in a dose-dependent manner in *Tpmt^+/+^*, *Tpmt^+/−^* and *Tpmt^−/−^* primary mouse astrocytes and that a low Tpmt phenotype predicted significantly higher sensitivity to TG than did a high Tpmt phenotype. We also found that TG exposure induced significantly more DNA damage in the form of single strand breaks (SSBs) and double strand breaks (DSBs) in primary astrocytes with low Tpmt versus high Tpmt. More interestingly, we found that *Tpmt^+/−^* astrocytes had the highest degree of cytotoxicity and genotoxicity (i.e., IC_50_, SSBs and DSBs) after TG exposure. We then used human glioma cell lines as model astroglial cells to represent high (T98) and low (A172) Tpmt expressers and found that A172 had the highest degree of cytoxicity and SSBs after TG exposure. When we over-expressed Tpmt in the A172 cell line, we found that TG IC_50_ was significantly higher and SSB's were significantly lower as compared to mock transfected cells. This study shows that low Tpmt can lead to greater sensitivity to thiopurine therapy in astroglial cells. When Tpmt deactivation at the germ-line is considered, this study also suggests that heterozygosity may be subject to the greatest genotoxic effects of thiopurine therapy.

## Introduction

**Figure 1:**
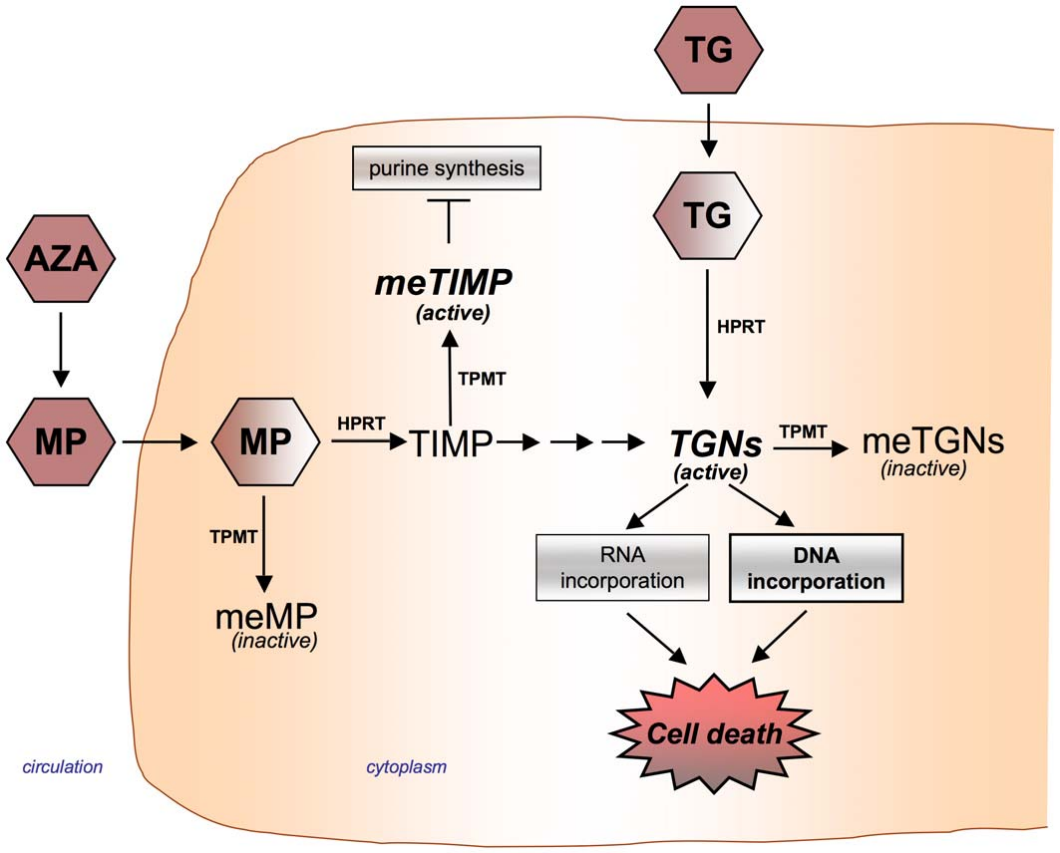
Thiopurine drug metabolism pathway. Azathioprine (AZA) is a prodrug of mercaptopurine (MP). Thioguanine (TG) and MP can be converted by hypoxanthine phosphoribosyl transferase (HPRT) to thioguanine nucleotide (TGNs) metabolites. MP can also be converted to the methylthioinosine monophosphate (meTIMP) by thiopurine methyltransferase (Tpmt) which inhibits purine synthesis; however, thioguanine bypasses the conversion to this metabolite. Thiopurines can be converted to inactive metabolites [i.e. methyl-mercaptopurine (meMP); methyl-thioguanine (meTG); and methyl-thioguanine nucleotides (meTGNs)] by Tpmt. TGN metabolites are incorporated into DNA and RNA leading to cell death. However, DNA incorporation is believed to be the primary mode of cytotoxicty.

Thiopurine methyltransferase (Tpmt) is a cytoplasmic enzyme responsible for catalyzing the S-methylation of aromatic and heterocyclic compounds and is the primary deactivating enzyme for thiopurine drugs [1]. Thiopurines are antimetabolite pro-drugs that require intracellular conversion to the active metabolites to exert their cytotoxic effects (Fig. 1) [2]. Alternatively, thiopurine activation is in competition with deactivation pathways primarily involving Tpmt. Tpmt has been studied extensively because of inter-patient variability in Tpmt protein expression owing to a few common genetic polymorphisms [3], [4], [5]. Deactivating polymorphisms in Tpmt can result in a trimodal population distribution in enzymatic activity. Inheritance of two functional alleles results in high protein activity (∼90% of individuals), heterozygosity leads to intermediate activity (∼10% of individuals), and inheritance of two dysfunctional alleles results in practically no protein activity (<1% of individuals) [6], [7]. Importantly, low Tpmt has been associated with life-threatening toxicities including cancer in patients receiving thiopurine therapy [8], [9], [10], [11], [12].

Over the past several decades, thiopurines [e.g. thioguanine (TG), mercaptopurine, and azathioprine] have been commonly used in the treatment of cancer, autoimmune disorders, and in organ transplant. In each of these patient populations there have been a number of reports of cancer development after thiopurine therapy to include acute myeloid leukemias, lymphomas, and cancers of the skin and brain [8], [13], [14], [15], [16], [17], [18], [19], [20], [21], [22], [23], [24]; although, the relevance of Tpmt status is not clear. However, in a study by Relling *et al* it was found that when patients were treated with antimetabolites (i.e. mercaptopurine, methotrexate) and prophylactic cranial irradiation as part of their antileukemic therapy, whether they developed a brain tumor depended on their Tpmt status [8], [24]. Patients who had a low Tpmt phenotype were more likely to develop a brain tumor as a late complication than those who had high Tpmt (42.9% versus 15.8% 10-year cumulative incidence, respectively) [8].

Tpmt is constitutively expressed in several tissues including blood, kidney, liver, and brain [25], [26], [27], [28], [29]. The association of Tpmt status with brain tumor risk after thiopurine exposure prompts the question of whether *Tpmt* genotypes can predict thiopurine drug phenotypes in astroglial cells. The relevance of Tpmt status to thiopurine-associated phenotypes in important brain cell populations has not been studied. Indeed, the first step to address the question of whether Tpmt plays any role in brain cancer risk after thiopurines is to determine whether Tpmt phenotypes in the brain are similar to those in other tissues. Thiopurine drug-associated genotoxicity has been linked to mutagenesis and transformative events [30], [31], [32]. We hypothesize that Tpmt deficiency can lead to greater genotoxicity (i.e. DNA damage) and cytotoxicity in astroglial cells after thiopurine exposure. To address this hypothesis we used primary astrocytes isolated from transgenic mice of each *Tpmt* genotype and performed *in vitro* studies to compare thiopurine-induced cytotoxicity and DNA damage between *Tpmt* genotypes. We also used established human glioma cell lines as model astroglial cells to validate the findings observed in primary mouse astrocytes.

## Results

### Characterization of *Tpmt^+/+^*, *Tpmt^+/−^*, and *Tpmt^−/−^* primary mouse astrocyte cultures

**Figure 2:**
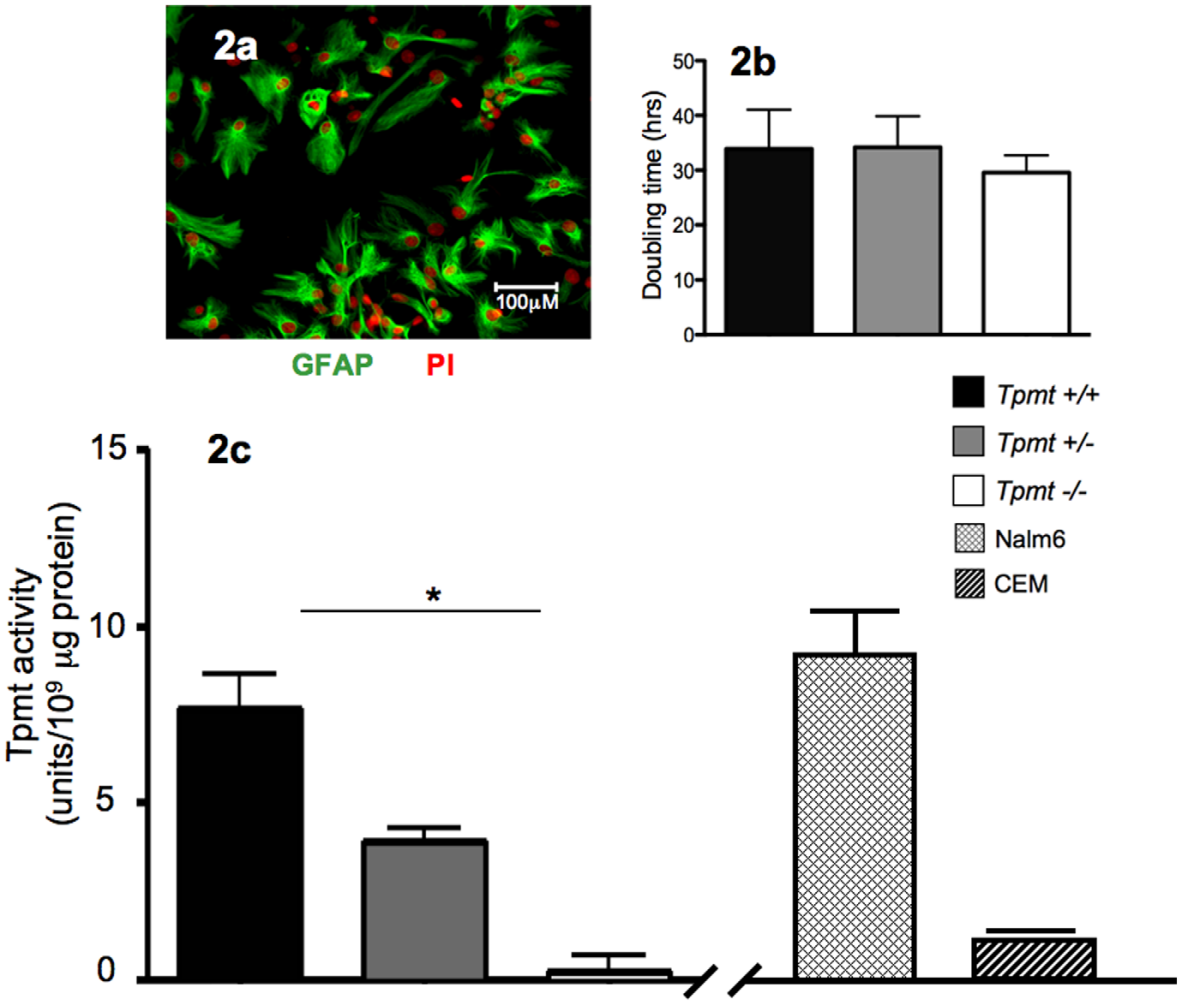
Characterization of *Tpmt^+/+^*, *Tpmt^+/−^*, and *Tpmt^−/−^* primary astrocyte cultures. (a) Micrograph image (200×) of primary astrocytes showing high astrocyte purity determined by GFAP (green) and propidium iodide (red) fluorescent staining. (b) There was no significant difference in growth rates between astrocyte cultures of each *Tpmt* genotype (p = 0.89). (c) The level of Tpmt protein activity was significantly different between the three *Tpmt* genotypes (p = 0.018); and levels were comparable to Nalm6 and CEM control lymphoblastoid cell lines. Bars represent the means; whiskers represent the standard deviation (±); * p<0.05.

The primary mechanism of thiopurine cytoxicity is believed to be through incorportation of TG nucleotide metabolites into DNA [2], [31], [33](Fig. 1), and therefore is dependent on the rate of cell proliferation. Hence, we first compared the doubling rates between *Tpmt^+/+^*, *Tpmt^+/−^*, and *Tpmt^−/−^* primary astrocyte cultures. Primary cultures were established from mouse cortices of each *Tpmt* genotype and astrocyte purity was confirmed by GFAP (glial fibrillary acid protein, an astrocyte marker) fluorescent staining (Fig. 2a). We found that there was no significant difference in growth rates between cultures of different *Tpmt* genotypes (Fig. 2b). The mean doubling rates for *Tpmt^+/+^*, *Tpmt^+/−^* and *Tpmt^−/−^* cultures were 33.9, 34.2, and 29.6 hours, respectively (p = 0.89). Next, we measured the level of Tpmt protein activity using a well-established radiochemical assay that measures the conversion of 6-mercaptopurine to 6-methylmercaptopurine using [14C] S-adenosyl-L-methionine (SAM) as a methyl donor [34]. In agreement with previous studies comparing protein activity in hematological cells of different *Tpmt* genotypes [5], [29], [35], protein activities in astrocytes were significantly different and were predicted by *Tpmt* genotype (Fig. 2c). *Tpmt^+/+^*, *Tpmt^+/−^*, *and Tpmt^−/−^* primary astrocytes displayed high (7.6 unit/10^9^ µg protein), intermediate (3.9 unit/10^9^ µg protein), and low (0.25 unit/10^9^ µg protein) levels of protein activity, respectively (p = 0.018). Based on these data, we next determined whether Tpmt could modulate the cytotoxic effects of TG in primary astrocytes.

### Comparison of thioguanine-associated cytotoxicity in *Tpmt^+/+^*, *Tpmt^+/−^*, and *Tpmt^−/−^* primary astrocyte cultures

**Figure 3:**
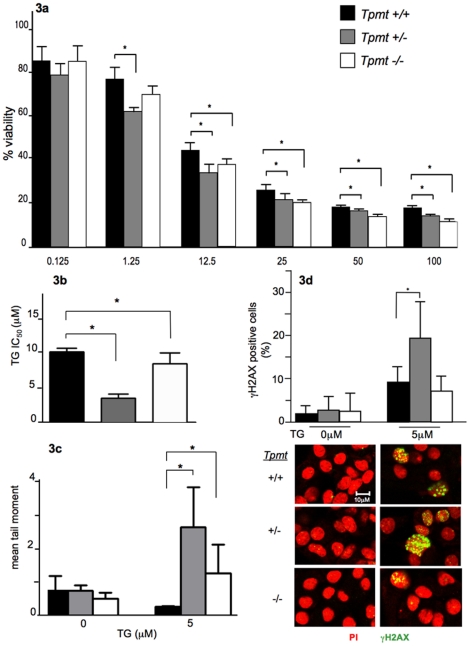
Comparison of thioguanine phenotypes in primary astrocyte cultures of each *Tpmt* genotype. (a) Primary astrocytes of each *Tpmt* genotype were exposed to thioguanine (TG) and cell viability was determined using the MTT assay. *Tpmt^+/−^* and *Tpmt^−/−^* astrocytes were significantly more sensitive to TG (12.5–100 µM) than *Tpmt^+/+^* (p<0.04). (b) IC_50_ values were significantly different between the three genotypes (p = 0.027) and *Tpmt^+/−^* astrocytes were the most sensitive. (c) The alkaline comet assay was used to compare TG-induced DNA damage between genotypes. *Tpmt^+/−^* and *Tpmt^−/−^* astrocytes had significantly higher mean comet tail moments than *Tpmt^+/+^* astrocytes (p = 0.033). (d) Immunoflourescence staining for γH2AX confirms IC_50_ and comet data. *Tpmt^+/−^* astrocytes had greater damage than *Tpmt^+/+^*(p = 0.03). Below are representative images of γH2AX staining for control treated (left) and TG treated (right) astrocytes. γH2AX foci stained in green; nuclei stained in red (Zeiss LSM 710 Laser Scanning Microscopes, 400× magnification). Bars represent the means; whiskers represent the standard deviation (±); * p<0.05.

To determine whether *Tpmt* could predict thiopurine cytotoxicity in primary astrocytes, the MTT colorimetric assay was employed to compare TG sensitivity between astrocytes of each *Tpmt* genotype. This assay measures cell viability by quantitating the extent at which metabolically active cells can reduce tetrazolium salt (MTT) to form purple formazan crystals. Astrocytes were exposed to escalating concentrations of TG (0.125, 1.25, 12.5, 25, 50, and 100 µM) for five days and then subjected to the MTT assay. Cell viability data at each TG concentration was used to calculate IC_50_ values. As expected, TG treatment resulted in cytotoxicity in a dose dependant manner (Figure 3a). Cell viability was significantly lower in *Tpmt^+/−^* and *Tpmt^−/−^* cultures at four concentrations of TG (12.5, 25, 50, and 100 µM) when compared to *Tpmt^+/+^* cultures (p<0.04). Interestingly, *Tpmt^+/−^* but not *Tpmt^−/−^* astrocytes were significantly more sensitive at 1.25 µM of TG than *Tpmt^+/+^* astrocytes (p = 0.004); and this finding was reflected in corresponding IC_50_ values. There was a 2.9 fold reduction in IC_50_ in *Tpmt^+/−^* (3.5 µM TG) versus a 1.2 fold reduction in *Tpmt^−/−^* (8.6 µM TG) when compared *Tpmt^+/+^* astrocytes (10.3 µM TG); and the *Tpmt^+/−^* and *Tpmt^−/−^* IC_50_ values were significantly different from *Tpmt^+/+^* (p<0.05) (Fig. 3b). Studies have shown that genotoxicity (i.e. DNA strand breaks) correlates with cytotoxicity after TG exposure [33], [36]. Hence, we investigated whether the degree of TG-induced DNA strand breaks was associated with the level of cytotoxicity in primary astrocytes.

### Comparison of the extent of DNA damage between *Tpmt^+/+^*, *Tpmt^+/−^*, and *Tpmt^−/−^* primary astrocytes

To address the question of whether the level of TG-induced cytotoxicity is correlated with DNA damage, we employed the alkaline comet assay. This assay allows for the quantitation of DNA damage [i.e. single strand breaks (SSBs), double strand breaks (DSBs) and alkali labile sites] by using fluorescent microscopy to visualize the extent of DNA migration from the nucleus of individual cells embedded in agarose [37]. We exposed astrocytes to 5 µM of TG for 72 hours. IC50 data was used to select a dose that would allow us to capture damage in each genotype. 72 hour time-point was selected to allow at least two cell doublings for TG incorporation and to capture cell damage prior to cell death. We observed a significant difference in DNA damage between the three genotypes as measured by mean comet tail moment. Both *Tpmt^+/−^* and *Tpmt^−/−^* astrocytes had a significantly greater degree of DNA damage than *Tpmt^+/+^* astrocytes (mean comet tail moment = 2.64, 1.26, and 0.4, respectively; p = 0.034) (Fig. 3c). In agreement with MTT studies, *Tpmt^+/−^* astrocytes were more sensitive to TG, as evidenced by a greater degree of DNA damage, than were *Tpmt^−/−^* astrocytes. Although the difference in DNA damage between *Tpmt^+/−^* and *Tpmt^−/−^* astrocytes was not significantly different (p = 0.083). There was no difference in DNA damage between *Tpmt* genotypes of control treated astrocytes.

Studies have shown that the predominant DNA lesions caused by TG are SSBs although some DSB lesions are formed [33]. Therefore, we then compared γH2AX (an established DSB marker) foci staining after TG exposure to determine the extent of the DNA lesions that are in the form of DSBs and whether this damage is different between *Tpmt* genotypes. Astrocytes were seeded on glass coverslips and exposed to either 0 or 5 µM of TG. We found that *Tpmt^+/−^* astrocytes had the greatest degree of foci staining (19.4%) compared to *Tpmt^+/+^* (9.3%), and *Tpmt^−/−^* (7.22%) (Fig. 3d). In agreement with comet data, *Tpmt^+/−^* was the most sensitive genotype. There was a significant difference between *Tpmt^+/−^ and Tpmt^+/+^* (p = 0.03) but no significant difference was observed between *Tpmt^−/−^ and Tpmt^+/+^* cells.

### Comparison of thioguanine phenotypes between human glioma cell lines with different Tpmt protein activities

**Figure 4:**
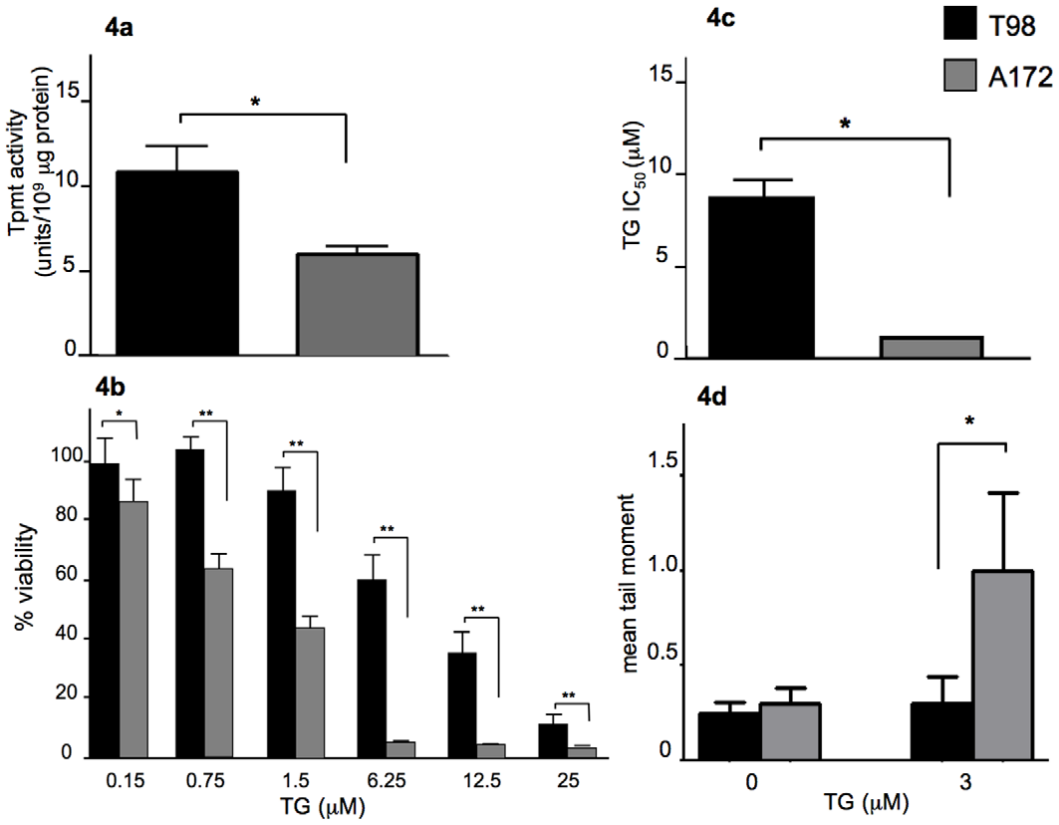
Assessment of Tpmt-associated phenotypes in model astroglial cell lines. Tpmt protein activity was measured in T98 and A172 glioma cell lines. (a) T98 displayed a significantly higher level of protein activity than A172 (p<0.05). (b) As observed in primary astrocyte cultures, low Tpmt protein activity is associated with greater cytotoxicity as determined by the MTT assay; (c) and reflected in IC_50_ values. A172 had a significantly more sensitive at all TG concentrations and lower IC_50_ than T98 (p<0.05). (d) The alkaline comet assay was used to compare TG-induced DNA damage. A172 displayed a significantly greater degree of TG-induced DNA strand breaks than T98 (p = 0.02) as measured by mean comet tail moment. Bars represent the means; whiskers represent the standard deviation (±); * p<0.05; ** p<0.005.

To determine whether our findings could be recapitulated in human cell lines, we used established human glioma cell lines as model astroglia and conducted MTT and comet experiments. We first identified cell lines with high and low Tpmt activity and compared TG-induced cytotoxicity and DNA damage between them. First, we measured Tpmt protein activity in four human glioma cell lines (T98, A172, U87, and LN18) to identify a high and low Tpmt expresser. We chose T98 (10.48 unit/10^9^ µg protein) and A172 (6.15 unit/10^9^ µg protein) as the high and low expressers, respectively (Fig. 4a). We then subjected T98 and A172 to the MTT assay to measure TG-induced cytotoxicity. Both cell lines were exposed to escalating concentrations of TG (0.15, 0.75, 1.5, 6.25, 12.5 and 25 µM) for five days. The cell viability was determined for each concentration and these data were used to calculate IC_50_ values. As predicted by Tpmt activity and in agreement with MTT studies performed in primary astrocytes, A172 had significantly lower cell viabilities at each TG concentration as compared to T98 (p<0.05) (Fig. 4b). The higher TG sensitivity of A172 was also reflected in the IC_50_ values. A172 displayed a 6.9 fold lower IC_50_ (1.2 µM) when compared to T98 (8.3 µM) (Fig. 4c).

Next, we employed the comet assay to compare DNA damage between T98 and A172 after exposing cells to 3 µM TG for 72 hours. In agreement with primary astrocyte data, a low level of Tpmt activity led to significantly more DNA damage compared to high activity (mean comet tail moment: A172 = 1, T98 = 0.3; p = 0.02) (Fig. 4d). DNA damage in control treated cells was not significantly different between the two cell lines.

### Comparison of thioguanine phenotypes between A172^mock^ and A172^Tpmt^ isogenic cell lines

**Figure 5:**
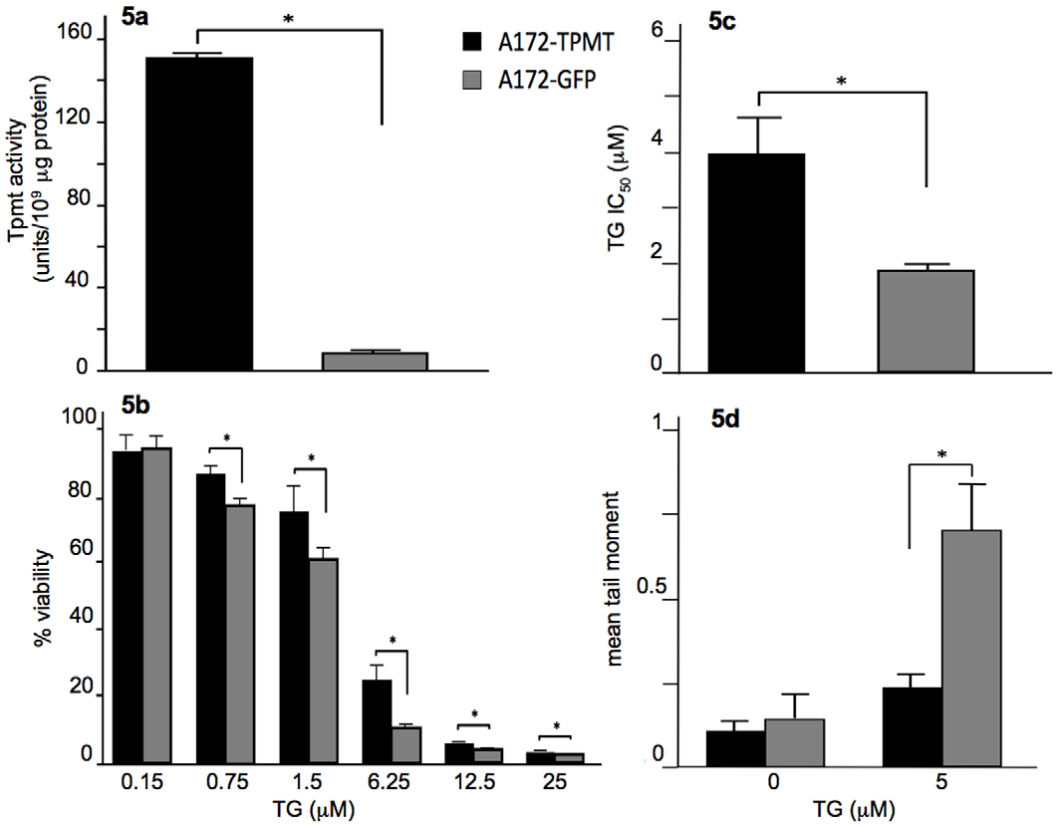
Comparison of Tpmt-associated phenotypes in A172 isogenic cells. Tpmt protein activity was measured in A172^mock^ and A172^Tpmt^ cell lines. (a) Tpmt protein activity was significantly higher in A172^Tpmt^ (p<0.05); additionally, (b) A172^Tpmt^ showed a significantly higher resistance to TG-induced toxicity when compared to A172^mock^ as determined by the MTT cell viability data (p<0.005) and (c) by IC_50_ values (p<0.05). (d) The alkaline comet assay was used to compare TG-induced DNA damage. A172^mock^ displayed a significantly higher DNA damage than A172^Tpmt^ (p = 0.03) as measured by mean comet tail moment. Bars represent the means; whiskers represent the standard deviation (±). * p<0.05.

To further validate our findings, TG phenotypes were compared using isogenic cell lines. A172 was the lowest Tpmt expresser, hence we over-expressed Tpmt in this cell line and compared TG-induced cytotoxicity and DNA damage between the mock transduced (A172^mock^) and Tpmt transduced (A172^Tpmt^) cell lines. We performed growth rate studies and found that there were no differences in doubling times between A172^Tpmt^ and A172^mock^ (28.5 and 29.6 hours, respectively; p = 0.38). The level of Tpmt protein activity in A172^Tpmt^ was significantly higher as compared to A172^mock^ (151.23 unit/10^9^ µg protein versus 9.47 unit/10^9^ µg protein, respectively; p<0.05) (Fig. 5a). When A172^mock^ and A172^Tpmt^ were exposed to escalating concentrations of TG for five days we found that A172^mock^ had significantly lower cell viabilities at 0.75, 1.5, 6.25, 12.5 and 25 µM of TG as compared to A172^Tpmt^ (p<0.005) (Fig. 5b). These findings were reflected in the A172^Tpmt^ IC_50_ value that was approximately two-fold higher than A172^mock^ (3.9 µM versus 1.9 µM, respectively; p<0.05)(Fig. 5c).

Next, we exposed A172^Tpmt^ and A172^mock^ to 5 µM of TG for 72 hours and compared the extent of DNA damage using the comet assay. We selected 5 µM based on the IC50 value of A172^Tpmt^. As expected, A172^mock^ had a significantly greater degree of DNA damage when compared to A172^Tpmt^ (mean comet tail moment: A172^mock^ = 0.7, A172^Tpmt^ = 0.24; p = 0.03) (Fig. 5d). DNA damage in control treated cells was not significantly different between the two cell lines (p = 0.35). These data support our findings observed in both the primary mouse astrocytes and the glioma cell lines (T98 and wild-type A172) that when Tpmt protein activity is low a greater degree of cytotoxicity and DNA damage can occur after TG exposure.

## Discussion

Tpmt is a cytoplasmic enzyme responsible for deactivating thiopurine drugs and a low Tpmt phenotype can lead to life-threatening hematological toxicities when thiopurines are given. Indeed, chronic administration of thiopurines has been linked to an increased risk of cancer including cancers of the brain [8], [15], [17], [22], [24]. Unfortunately, not all patient populations that are treated with thiopurines are evaluated for their Tpmt status prior to initiating thiopurine therapy; hence, the importance of Tpmt to thiopurine-associated cancer risk is not clear. This fact coupled with the fact that Tpmt deficiency is relatively rare across populations, makes it difficult to assess the importance of Tpmt status to thiopurine therapy-related cancer risk. However, in a report by Relling *et al* whether patients developed a brain tumor as a late complication post-antileukemic therapy depended on their Tpmt status [24]. In this report, patients who were determined to be at high risk for treatment relapse received prophylactic cranial irradiation concurrent with antimetabolite therapy; and those patients with a low Tpmt had a surprisingly high incidence of brain tumors as a late complication. Indeed, ionizing radiation in itself is a risk factor for brain tumorigenesis. However, in this report, patients with low Tpmt had a 42.9% incidence of brain tumors compared to 15.8% for those with high Tpmt.

Astrocytomas are the most common brain tumor type that has been reported in patients after thiopurine therapy [8], [15], [22], [24]. The importance of Tpmt status to thiopurine-associated phenotypes in astroglial cells has never been studied. We assessed Tpmt and TG phenotypes in astroglial cells using primary mouse astrocytes of each *Tpmt* genotype and human glioma cells of different Tpmt phenotypes. In this study we have shown that TG induces cytotoxicity in a dose-dependent manner in astroglial cells; and cells with a low Tpmt phenotype (*Tpmt^+/−^* and *Tpmt^−/−^* astrocytes and A172^mock^) were significantly more sensitive. We also showed that TG exposure induced more DNA damage in the form of SSBs in astroglial cells with low Tpmt than in cells with high Tpmt. DNA damage in the form of DSBs was significantly different between astroglial cells with low and high Tpmt. SSBs have been shown to be the primary DNA lesion induced by thiopurine drugs [33].

Interestingly, we found that low but not the complete absence of Tpmt sensitized primary astrocytes to a higher degree of cytotoxicity and genotoxicity (i.e. IC_50_, SSBs and DSBs) after TG exposure. This discordance could possibly be explained by the induction of a compensatory mechanism that deactivates thiopurines with the complete loss of Tpmt. Another possible explanation could be a defect in the mismatch repair (MMR) system's ability to recognize DNA-TG nucleotide lesions. The MMR system is responsible for recognizing DNA mis-pairing caused by DNA-TG nucleotides and the subsequent signaling for cell death [38], [39], [40], [41]. The latter possibility suggests that astroglial cells with a defective Tpmt could have DNA lesions persisting in the genome that could potentially lead to mutagenesis and cancer. Indeed, some studies suggest that heterozygous children develop brain tumors and other cancers more often than children with mutant *Tpmt*. However, the frequency of heterozygous or mutant *Tpmt* is very low making it is difficult to make any definitive conclusions [24], [42]. It would be interesting to investigate whether Tpmt status influences the extent of TG nucleotide incorporation into DNA and damage recognition by the MMR system as well as whether ionizing irradiation alone or combined with thiopurines results in similar findings.

In summary, chronic thiopurine therapy has been associated with the development of brain cancer and Tpmt status has been linked to this risk. We found that Tpmt is an important factor for thiopurine drug-induced cytotoxicity and genotoxicity in astroglial cells. Even though glioma cells can exhibit a number of genetic alternations that could contribute to the phenotypes observed, our study shows that Tpmt is indeed an important determinant of thiopurine toxicity. Low Tpmt can increase the level of TG-induced genotoxic damage in both normal and in neoplastic astroglial cells. Indeed, the observations that glioma cells can have variable degrees of Tpmt protein activity and that low protein activity can cause cells to be more sensitive to TG poses the question of whether thiopurines may potentially have a therapeutic role in low Tpmt expressing glial tumors. *In vitro* studies have shown that thiopurine drugs are associated with mutagenic events in a variety of cell lines [30], [36]. It is possible that the DNA damage caused by low Tpmt function can ultimately contribute to transformative events over time. One study found that Tpmt was not an important factor for cancer risk after thiopurine therapy [42]; however in this study only Tpmt genotype was determined which may have excluded patients with discordance between Tpmt genotype and phenotype. Individuals who are wild-type for Tpmt can display a wide variation in their Tpmt phenotype [43]. Further studies are needed to understand the importance of Tpmt in the brain when thiopurines are given. Whether theses findings can be replicated *in vivo* and whether factors important to DNA repair mechanisms are responsible for the TG phenotypes described in this study should also be investigated.

## Materials and Methods

### Animals

The Tpmt knockout mice were a generous gift of Dr. Mary Relling (St. Jude Children's Research Hospital) and the generation of these mice has been described previously [35]. The *Tpmt^+/−^*, and *Tpmt^−/−^* mice were indistinguishable from *Tpmt^+/+^* mice by appearance, life span, and organ histology (i.e. brain, liver, spleen, intestine, thymus, and lymph nodes). Animals were maintained in accordance to NIH guidelines for the care and use of laboratory animals and were housed in an AAALAC accredited facility. The University of Tennessee Health Science Center Institutional Animal Care and Use Committee approved all animal procedures.

### Primary astrocyte cultures and cell lines

Astrocyte cultures were established from 2–5 day old pups of each *Tpmt* genotype. The DNA extracted from tails snips was used to confirm *Tpmt* genotype. A dissecting microscope was used to remove the meninges and hippocampus and the cortices were mechanically dissociated. Subsequently, cultures were established and maintained in Dulbecco's modified Eagle's medium/Ham's F-12 50/50 mix (Cellgro) supplemented with 10% fetal bovine serum, 2 mM L-glutamine, 100 IU/ml penicillin, 100 µg/ml streptomycin and 20 ng/ml epidermal growth factor (Millipore) and grown in Primaria flasks, (BD biosciences). Cells were refed with 20 ng/ml epidermal growth factor after three days of incubation, and the media was changed after five days. At passage 2, three individual primary cultures of the same genotype were pooled and seeded for experiments. Only cultures with greater than 80% astrocyte purity as determined by GFAP immunohistochemistry were included in experiments.

The T98 and A172 cell lines were obtained from the American Type Culture Collection (Manassas, VA, USA). T98 was grown in Minimum Essential Medium Eagle (Cellgro), supplemented with 10% fetal bovine serum, 2 mM L-glutamine, 0.1 mM nonessential amino acids, 1 mM sodium pyruvate, 100 IU/ml penicillin, and 100 µg/ml streptomycin. A172 was grown in Dulbecco's Modified Eagle's Medium (Cellgro), supplemented with 10% fetal bovine serum, 2 mM L-glutamine, 100 IU/ml penicillin, and 100 µg/ml streptomycin. All cell cultures were maintained at 37°C in a 5% CO_2_ humidified atmosphere.

### Lentiviral transduction

Human Tpmt cDNA was obtained by digesting pCMV6-Tpmt plasmid (Origene) at BamHI and EcoRI sites. Tpmt cDNA was then amplified by PCR, purified through agarose gel electrophoresis, and subcloned into BamHI and EcoRI sites of pLenti-CMV-pgk-puro vector (Viral vector core, UTHSC). The plenti-Tpmt-pgk-puro and plenti-GFP-pgk-puro lentivial vectors were produced by packaging in 293T cells [44]. The lentiviral vectors were used to transduce the A172 cell line in the presence of 6 µg/mL polybrene (Sigma). Transduction efficiency was monitored by GFP expression using fluorescent microscope. The mock- (A172^mock^) and Tpmt-transduced (A172^Tpmt^) cell lines were seeded for experiments at comparable passage numbers and Tpmt activity was measured prior to the experiments.

### Cell proliferation comparison

The doubling rates for primary astrocyte cultures were determined and used as an index of cell proliferation. At passage two, 75,000 cells/well were seeded in duplicate 6-well plates. Every 24 hours the cells from one well of each plate were harvested using 0.05% Trypsin-EDTA (GIBCO) and viable cells were counted by trypan blue exclusion for four consecutive days. Doubling rates were calculated using the following formula: Doubling time (hours) = (T2-T1)/[log2×(Log N-Log N_0_)]. Where, T2 = Harvesting time, T1 = Initial time, N = Final cell concentration, and N_0_ = Initial cell concentration. The mean doubling rate was calculated from replicate experiments using two different pooled cultures established on different days.

### TPMT activity assay

Primary mouse astrocyte and human glioma cell lines were sonicated using a probe sonicator (Masonix XL-2000 series) prior to performing the Tpmt activity assay. The level of Tpmt activity was determined using a pre-established non-chelated radiochemical assay [26], [34] with slight modifications. Tpmt activity was quantitated by measuring the extent of conversion of 6-mercaptopurine (Sigma) to radioactively labeled 6-methylmercaptopurine with [14C] S-adenosyl-L-methionine (SAM) (Perkin-Elmer). The protein concentration of each lysate was measured prior to performing the assay and was used to calculate Tpmt activity. The mean activities for each genotype were calculated using data from triplicate inter-day studies of three separate pooled cultures per *Tpmt* genotype.

### MTT assay

Cell viability was measured using 3-(4,5-dimethylthiazol-2-yl)-2,5-diphenyltetrazolium bromide (Sigma). Briefly, 1000 cells were seeded per well in 96-well plates. Plates were pre-coated with poly-L-ornithine (Sigma) and Laminin (Invitrogen) for primary astrocytes to facilitate cell attachment. A fifteen millimolar stock solution of TG (Sigma) was prepared by dissolving TG in 0.1 N NaOH. Cells were allowed to attach over night and TG or vehicle control was added to the appropriate wells. After five days of drug exposure, MTT was added and allowed to incubate for 3 hours. Afterwards, the media was carefully aspirated and the purple formazan crystals were dissolved in 100 µl DMSO (Fisher). Absorbance was measured at wavelength of 570/690 nm using a FLx800 fluorescence microplate reader (BioTek Instruments, Inc.).

### Alkaline comet assay

Techniques used to perform the comet assay were adopted from the laboratory of Dr. Peter Mckinnon (St. Jude Children's Research Hospital) [45]. Briefly, 50,000 primary astrocyte or 30,000 human astroglial cells were seeded in Primaria 24-well plates (BD Biosciences) or in Falcon 24-well plates (BD Biosciences), respectively. Cells were allowed to attach overnight and then exposed to TG for three days. After three days of drug treatment, cells were harvested and counted to prepare a 3×10^5^ cells/ml suspension in PBS. The cell suspension was mixed with 1.2% ultra-pure low melting point agarose (Invitrogen) and casted onto chilled fully frosted glass slides (Fisher) pre-coated with 0.6% agarose (Bio-Rad). The cells were then lysed for 1.5 hours at 4°C, in lysis buffer (100 µM EDTA, 2.5 M NaCl, 10 mM Tris, 1.3% triton X-100 and 3.3% DMSO). The slides were washed twice with distilled water and incubated for 45 minutes at 4°C in electrophoresis buffer (1 mM EDTA, 50 mM NaOH and 1% DMSO). Electrophoresis was conducted at 12 V and ∼90 mA at 4°C for 25 minutes. Slides were incubated for one hour in 400 mM Tri-HCl neutralization buffer followed by a 20 minute incubation in SYBR green I (Sigma). Images were captured using a Nikon eclipse TE300 fluorescent microscope. All experiments were replicated using two different sets of pooled cultures. The extent of DNA damage was expressed as the comet tail moment (the amount and distribution of DNA in the tail). The comet tail moment was measured in a minimum of 60 cells using Cometscore version 1.5 (Tri Tek corp).

### Immunohistochemistry

Primary astrocytes were grown on poly-D-lysine chamber slides (BD Biocoat) for GFAP staining (to confirm astrocyte purity) or on glass coverslips and treated with 0 or 5 µM of TG for γH2AX staining. Cells were fixed with 4% PFA/PBS for 10 minutes, permeabilized with 0.5% Triton X-100/PBS for 5 minutes. Cells were then washed with PBS and blocked with 3% BSA/PBS for one hour. Cells were then immunostained with either anti-GFAP (1∶500 in PBS, Sigma) for two hours or anti-γH2AX (1∶1000 in 3%BSA, Milipore) for one hour. Subsequently, cells were washed with PBS and incubated for one hour with in Cy2-conjugated donkey anti-mouse secondary antibody (Jackson ImmunoResearch, 1∶250 in PBS containing 2% donkey serum and 0.1% Triton X-100) for GFAP or with donkey anti-mouse secondary antibody labeled with Alexa 488 (In vitrogen, 1∶800 in 3% BSA) for one hour. Finally, vectashield mounting media (Vectorlabs) containing propidium iodide was used to stain nuclei. GFAP slides were visualized and images were captured using Nikon eclipse TE300 fluorescent microscope. Images were captured for γH2AX stained slides using a LSM-710 confocal microscope (Zeiss). Images were analyzed for γH2AX foci by counting a minimum of 100 cells per genotype. Cells containing fewer than five foci were excluded to normalize for baseline staining observed in control treated cells. Cells with “pan-staining” were considered to have more than 5 foci per cell. Hence, the percent of cells with γH2AX foci staining represents the percent of cells with greater than five foci per cell.

### Statistical analysis

Differences in cytotoxicity and DNA damage among all three groups were compared using Kruskal-Wallis analysis of ranks. Mann-Whitney *U* test was used when only two groups were compared. Significance was declared when p<0.05. All statistical analyses were performed using SAS 9.2 (SAS Institute Inc., Cary, NC).
